# Plasma NGAL levels in stable kidney transplant recipients and the risk of allograft loss

**DOI:** 10.1093/ndt/gfad226

**Published:** 2023-10-19

**Authors:** Jutta S Swolinsky, Ricarda M Hinz, Carolin E Markus, Eugenia Singer, Friederike Bachmann, Fabian Halleck, Susanne Kron, Marcel G Naik, Danilo Schmidt, Martin Obermeier, Pimrapat Gebert, Geraldine Rauch, Siegfried Kropf, Michael Haase, Klemens Budde, Kai-Uwe Eckardt, Timm H Westhoff, Kai M Schmidt-Ott

**Affiliations:** Department of Nephrology and Medical Intensive Care, Charité – Universitätsmedizin Berlin, corporate member of Freie Universität Berlin and Humboldt-Universität zu Berlin, Department of Nephrology and Medical Intensive Care, Berlin, Germany; Max Delbrück Center for Molecular Medicine, Berlin, Germany; Department of Nephrology and Medical Intensive Care, Charité – Universitätsmedizin Berlin, corporate member of Freie Universität Berlin and Humboldt-Universität zu Berlin, Department of Nephrology and Medical Intensive Care, Berlin, Germany; Max Delbrück Center for Molecular Medicine, Berlin, Germany; Department of Nephrology and Medical Intensive Care, Charité – Universitätsmedizin Berlin, corporate member of Freie Universität Berlin and Humboldt-Universität zu Berlin, Department of Nephrology and Medical Intensive Care, Berlin, Germany; Max Delbrück Center for Molecular Medicine, Berlin, Germany; Department of Nephrology and Medical Intensive Care, Charité – Universitätsmedizin Berlin, corporate member of Freie Universität Berlin and Humboldt-Universität zu Berlin, Department of Nephrology and Medical Intensive Care, Berlin, Germany; Max Delbrück Center for Molecular Medicine, Berlin, Germany; Department of Nephrology and Medical Intensive Care, Charité – Universitätsmedizin Berlin, corporate member of Freie Universität Berlin and Humboldt-Universität zu Berlin, Department of Nephrology and Medical Intensive Care, Berlin, Germany; Department of Nephrology and Medical Intensive Care, Charité – Universitätsmedizin Berlin, corporate member of Freie Universität Berlin and Humboldt-Universität zu Berlin, Department of Nephrology and Medical Intensive Care, Berlin, Germany; Department of Nephrology and Medical Intensive Care, Charité – Universitätsmedizin Berlin, corporate member of Freie Universität Berlin and Humboldt-Universität zu Berlin, Department of Nephrology and Medical Intensive Care, Berlin, Germany; Department of Nephrology and Medical Intensive Care, Charité – Universitätsmedizin Berlin, corporate member of Freie Universität Berlin and Humboldt-Universität zu Berlin, Department of Nephrology and Medical Intensive Care, Berlin, Germany; Berlin Institute of Health at Charité – Universitätsmedizin Berlin; Department of Nephrology and Medical Intensive Care, Charité – Universitätsmedizin Berlin, corporate member of Freie Universität Berlin and Humboldt-Universität zu Berlin, Department of Nephrology and Medical Intensive Care, Berlin, Germany; Medizinisches Infektiologiezentrum (MIB), Berlin, Germany; Berlin Institute of Health at Charité – Universitätsmedizin Berlin; Charité – Universitätsmedizin Berlin, corporate member of Freie Universität Berlin and Humboldt-Universität zu Berlin, Institute of Biometry and Clinical Epidemiology; Berlin Institute of Health at Charité – Universitätsmedizin Berlin; Charité – Universitätsmedizin Berlin, corporate member of Freie Universität Berlin and Humboldt-Universität zu Berlin, Institute of Biometry and Clinical Epidemiology; Institute of Biometry and Medical Informatics, Otto-von-Guericke University Magdeburg, Magdeburg, Germany; Medical Faculty, Otto-von-Guericke University Magdeburg, Magdeburg, Germany; Department of Nephrology and Hypertension, Hannover Medical School, Hannover, Germany; Diaverum Renal Services, MVZ Potsdam, Potsdam, Germany; Department of Nephrology and Medical Intensive Care, Charité – Universitätsmedizin Berlin, corporate member of Freie Universität Berlin and Humboldt-Universität zu Berlin, Department of Nephrology and Medical Intensive Care, Berlin, Germany; Department of Nephrology and Medical Intensive Care, Charité – Universitätsmedizin Berlin, corporate member of Freie Universität Berlin and Humboldt-Universität zu Berlin, Department of Nephrology and Medical Intensive Care, Berlin, Germany; Medical Department I, Marien Hospital Herne, Universitätsklinikum der Ruhr-Universität Bochum, Bochum, Germany; Department of Nephrology and Medical Intensive Care, Charité – Universitätsmedizin Berlin, corporate member of Freie Universität Berlin and Humboldt-Universität zu Berlin, Department of Nephrology and Medical Intensive Care, Berlin, Germany; Max Delbrück Center for Molecular Medicine, Berlin, Germany; Department of Nephrology and Hypertension, Hannover Medical School, Hannover, Germany

**Keywords:** biomarkers, calprotectin, graft survival, kidney transplantation, NGAL

## Abstract

**Background:**

The objective of this study was to investigate the utility of neutrophil gelatinase-associated lipocalin (NGAL) and calprotectin (CPT) to predict long-term graft survival in stable kidney transplant recipients (KTR).

**Methods:**

A total of 709 stable outpatient KTR were enrolled >2 months post-transplant. The utility of plasma and urinary NGAL (pNGAL, uNGAL) and plasma and urinary CPT at enrollment to predict death-censored graft loss was evaluated during a 58-month follow-up.

**Results:**

Among biomarkers, pNGAL showed the best predictive ability for graft loss and was the only biomarker with an area under the curve (AUC) > 0.7 for graft loss within 5 years. Patients with graft loss within 5 years (*n* = 49) had a median pNGAL of 304 [interquartile range (IQR) 235–358] versus 182 (IQR 128–246) ng/mL with surviving grafts (*P *< .001). Time-dependent receiver operating characteristic analyses at 58 months indicated an AUC for pNGAL of 0.795, serum creatinine–based Chronic Kidney Disease Epidemiology Collaboration estimated glomerular filtration rate (eGFR) had an AUC of 0.866. pNGAL added to a model based on conventional risk factors for graft loss with death as competing risk (age, transplant age, presence of donor-specific antibodies, presence of proteinuria, history of delayed graft function) had a strong independent association with graft loss {subdistribution hazard ratio (sHR) for binary log-transformed pNGAL [log2(pNGAL)] 3.4, 95% confidence interval (CI) 2.24–5.15, *P *< .0001}. This association was substantially attenuated when eGFR was added to the model [sHR for log2(pNGAL) 1.63, 95% CI 0.92–2.88, *P *= .095]. Category-free net reclassification improvement of a risk model including log2(pNGAL) in addition to conventional risk factors and eGFR was 54.3% (95% CI 9.2%–99.3%) but C-statistic did not improve significantly.

**Conclusions:**

pNGAL was an independent predictor of renal allograft loss in stable KTR from one transplant center but did not show consistent added value when compared with baseline predictors including the conventional marker eGFR. Future studies in larger cohorts are warranted.

## Graphical Abstract

**Graphical Abstract:**
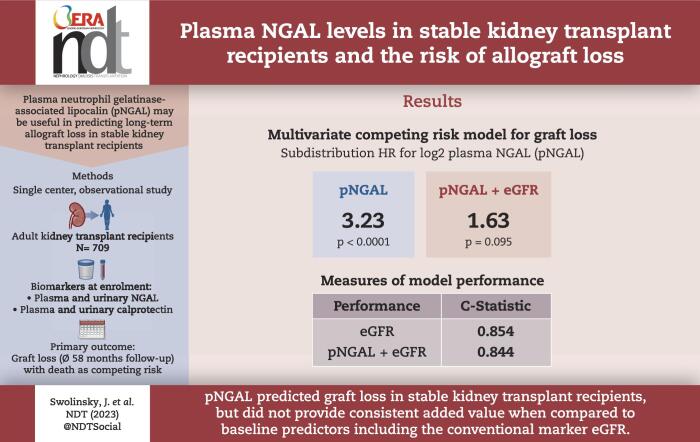

KEY LEARNING POINTS **What was known:**

• Neutrophil gelatinase-associated lipocalin (NGAL) and calprotectin are biomarkers of tubular injury that have been predominantly studied in the setting of acute kidney injury and short-term outcomes such as delayed graft function. However, little is known about the possibility that these biomarkers might also predict subclinical kidney injury in stable kidney transplant recipients and thereby predict adverse long-term outcomes.

**This study adds:**

• In this prospective study, we investigated the predictive value of urinary and plasma neutrophil gelatinase-associated lipocalin and calprotectin regarding graft loss during 5 years of follow-up in 709 stable kidney transplant recipients with death as competing risk. pNGAL was strongly and independently associated with graft failure but did not show consistent added value when compared to baseline predictors including the conventional marker eGFR.

**Potential impact:**

• The additional determination of plasma neutrophil gelatinase-associated lipocalin to conventional clinical parameters like serum creatinine and proteinuria measured in stable outpatient kidney transplant recipients might be of value in the prediction of graft survival. Future studies in larger cohorts are warranted.

## INTRODUCTION

Chronic loss of function with premature kidney transplant failure represents the greatest challenge in kidney transplantation. Progressive improvement in immunosuppressive therapy and medical follow-up resulted in an improved graft survival during the last three decades, especially between 1988 and 1996 [1]. However, this success is mostly limited to the first 3 years after transplantation [2, 3]. Progress in improving graft survival in Europe since 2000 has been significantly slower than in previous decades, especially with regard to the first 5 years after transplantation [4]. International data report a 5-year graft survival of 86% for grafts from living donors and 76% for grafts from deceased donors since 2000 [5]. The cause of graft loss is often multifactorial. A recently published study investigated the reasons for death-censored kidney allograft failure among 1477 kidney transplant recipients (KTR) during a 20-year follow-up. The most frequent overall causes leading to graft failure were intercurrent medical events (e.g. cardiovascular events, infections, 36.3%), followed by T cell–mediated rejection (34%) and antibody-mediated rejection (30.7%) [6].

KTR are at risk for subclinical and manifest episodes of acute kidney injury (AKI) predisposing them to functional decline [7, 8]. It has recently been demonstrated that over the lifetime of a graft, multiple acute kidney injuries ultimately contribute to graft loss [6]. Currently, graft integrity is monitored by measurements of serum creatinine and proteinuria. Kidney biopsies are performed if these markers suggest relevant transplant pathology. However, creatinine and proteinuria lack sensitivity for detecting structural or functional changes and periods of “subclinical” kidney injury may escape detection.

One of the most intensively studied biomarkers in the context of kidney injury is neutrophil gelatinase-associated lipocalin (NGAL) [9]. NGAL is a 25-kDa marker of tubular damage that is freely filtered in the glomerulus and reabsorbed in the proximal tubule [10]. During AKI, proximal tubule reabsorption of systemically synthetized NGAL is impaired, and NGAL production by the kidney tubules is increased, leading to higher urine and plasma NGAL levels [11–13]. In several clinical settings, NGAL allows an early diagnosis of AKI, including perioperative AKI [12, 14], sepsis-associated AKI [15], contrast-induced AKI [16] and critical illness–associated AKI in ICU patients [17, 18], as well as AKI in patients in the emergency room [19–21] and AKI following kidney transplantation [22–25]. Moreover, urinary NGAL (uNGAL) differentiates between prerenal azotemia and intrinsic kidney injury [14, 21].

Calprotectin (CPT) is another marker of kidney damage that has also been widely studied. It is a 24-kDa calcium-binding complex consisting of the two proteins S100A8 and S100A9 [26] produced by renal collecting duct epithelial cells in response to damage [27]. Measuring CPT levels in the urine distinguishes between prerenal and intrinsic AKI, including in KTRs [28–30].

Most of the biomarker studies in KTRs were conducted at timepoints of presumed acute transplant injury (e.g. shortly after transplantation) and focused on short-term outcomes like delayed graft function (DGF) [31]. Little is known about NGAL and CPT in the chronic phase after kidney transplantation and their role in detecting ongoing subclinical injury and in predicting long-term outcomes.

In this study, we addressed the potential of plasma and urinary NGAL as well as CPT levels in stable KTR to predict graft loss during the following 5 years.

## MATERIALS AND METHODS

All adult KTR, capable of giving consent, with a transplant age of at least 2 months, who were regularly visiting the kidney transplant outpatient clinic of Charité Universitätsmedizin Berlin Campus Mitte for follow-up care, were considered for study inclusion. A total of 798 potential study participants were identified between April and September 2013. Patients with a history of malignancy within the past 5 years were excluded. A stable clinical condition, defined as the absence of indication for inpatient admission or infection parameters, a stable graft function, defined as a serum creatinine that did not differ >0.3 mg/dL from the previous three values and an estimated glomerular filtration rate (eGFR) calculated by serum creatinine–based Chronic Kidney Disease Epidemiology Collaboration (CKD-EPI_sCr_) equation >15 mL/min/1.73 m^2^, were requirements for study inclusion. After consent, plasma and urinary samples for biomarker determination were taken. Routine laboratory values were analyzed in the hospital's local laboratory as part of routine follow-up care and captured together with the clinical outcome in the TBase database [32]. Patient characteristics (including demographics, comorbidities, transplant characteristics, biomarker samples, routine laboratory values) were collected after consent at the time of study inclusion. Follow-up period started at enrollment.

### Informed consent and ethics

The Charité University Ethics Committee approved the study (EA1/320/12) and written informed consent was obtained at the time point of enrollment. The study was conducted in accordance with Declaration of Helsinki guidelines. The study protocol and primary endpoint was registered prospectively at the Charité University Ethics Committee.

### Biomarker measurements

Urinary and plasma samples were centrifuged 10 min at 3500 revolutions. The supernatant was pipetted and frozen (−80°C) within 6 h after sample collection until assessment September 2014. NGAL was determined using the NGAL Test™ Reagent Kit (Bioporto^®^, Gentofte, Denmark), a particle-enhanced turbidimetric immunoassay according to the manufacturer's protocol. The CPT concentration was determined using the IDK^®^ Calprotectin ELISA Kit (IDK^®^ Calprotectin, catalog number K 6935 and K 6928; Immundiagnostik AG, Bensheim, Germany) which is based on a sandwich enzyme-linked immunosorbent assay technique. Serum creatinine (sCr) concentration was obtained through the Jaffé method.

eGFR was calculated using sCr-based CKD-EPI formula from 2009 (eGFR CKD-EPI_sCr_) [33].

### Follow-up and endpoint definition

Patients were followed up for a predefined primary endpoint of death-censored graft loss for approximately 5 years from enrollment (April–September 2013) until April 2018. Graft loss was defined as reinitiation of dialysis, allograft-nephrectomy or retransplantation. Follow-up time was defined as duration from inclusion until graft loss, death or study end.

### Statistical analysis

Statistical analyses were conducted with IBM SPSS statistics 24, Stata (version IC 15.1), SAS (version 9.4) and R (version 3.5.1). Continuous data were presented as median and interquartile range (IQR) or mean and standard deviation (SD) as appropriate. Testing for group differences between two groups with respect to metric, non-normally distributed, variance-inhomogeneous variables was conducted using the Mann–Whitney U test. Categorical variables were compared by χ^2^ test. Distribution of biomarker, sCr and eGFR CKD-EPI_sCr_ concentrations are presented as Box plots (GraphPad Prism version 8.3.0, GraphPad software, San Diego, CA, USA).

Time-dependent receiver operating characteristics (*time*ROC) for graft loss with death as competing risk and controls defined as subjects that are free of any event were conducted to evaluate the prognostic value of the plasma and urinary biomarkers and kidney function markers regarding the outcomes after a 3, 4 and 5 years follow-up [34, 35]. Markers with a *time*ROC area under the curve (AUC) >0.7 for all follow-up lengths were considered acceptable discriminators and subsequent analyses focused on these variables only [plasma NGAL (pNGAL), eGFR].

The primary analysis for graft loss has been performed with Fine and Gray's proportional sub-hazard model (SAS, PROC PHREG) with death as competing risk [36].

A multivariate base model adjusted for risk factors for graft loss was used as a reference model for the assessment of the additional biomarkers. The predictors chosen for adjustment in the multivariate model were selected based on traditional risk factors associated with graft loss, including proteinuria, presence of donor-specific antibodies (DSA), history of DGF, transplant age and recipient age [6]. pNGAL and eGFR were each added to the multivariate base model. Three model variations were presented to explore the mutual influence on the predictive value of each marker: pNGAL-assisted model, eGFR-assisted model and pNGAL- and eGFR-assisted base model. pNGAL and eGFR were used on a binary logarithmic scale (log2). We further performed sensitivity analyses with log2(pNGAL) and log2(eGFR) replaced by spline transformations retaining the other variables of the base model.

In the selection of splines, we omitted an optimization by usual fit criteria to prevent a weakening of the final regression tests. However, we changed the original version (default version in SAS, PROC PHREG) with B-splines and three equidistant knots and splines of degree 3 to splines of degree 2 because of numerical instabilities in the imputation and bootstrap repetitions (see below) and large confidence intervals (CIs). For the same reason, we reduced the number of knots for eGFR to one.

The graphical presentation of spline regressions for graft loss was created using IBM SPSS. A proportional hazards model with death considered as censoring and omitting the incomplete data vectors was considered for testing the proportional hazards assumption in the global correlation test with weighted Schoenfeld residuals.

Missing data occurring in the three categorical variables of the base model were completed by multiple imputation by the fully conditional specification method based on a logistic regression model with age at enrollment, age of transplant, gender, eGFR, proteinuria, DSA status, DGF, pNGAL, plasma CPT (pCPT), loss of transplant, death for other reasons and the cumulative incidence function from a model without covariables as independent variables (SAS PROC MI). The proportion of missing values was 1%–2% for donation type and proteinuria, and 4% for DGF and positive DSA status. Ten imputed datasets were generated, and the results were combined using Rubin's rule [37] with SAS PROC MIANALYZE.

C-indices were calculated according to Longato *et al*. [38] to assess and compare model discrimination power of biomarker assisted models. An additional bias adjustment according to Geroldinger *et al*. [39] and the determination of CIs for the C-indices and their difference have been accomplished using bootstrapping with 20 replications. In this process, the bias is estimated as difference between the C-indices derived from bootstrap samples and the C-indices of the original data when applied to regression rule derived from the bootstrap sample.

Category-free Net Reclassification Improvement (NRI) and NRI based on two categories (*a priori*–defined risks of 0%–5% and >5%) was calculated to assess incremental improvement in risk reclassification according to a proposal of Pencina *et al*. for survival data [40]. CIs for both versions are derived with bootstrapping (20 replications).

Univariable linear regression analyses were performed with binary log-transformed pNGAL [log2(pNGAL)] as the dependent variable. Normality of the residuals was evaluated by visual inspection of QQ-plots. Variables were transformed by binary logarithm if necessary to reach assumptions for linear regression. R^2^ and Spearman correlations were presented additionally to assess linear and non-parametric correlation.

## RESULTS

### Baseline patient characteristics

**Figure 1:**
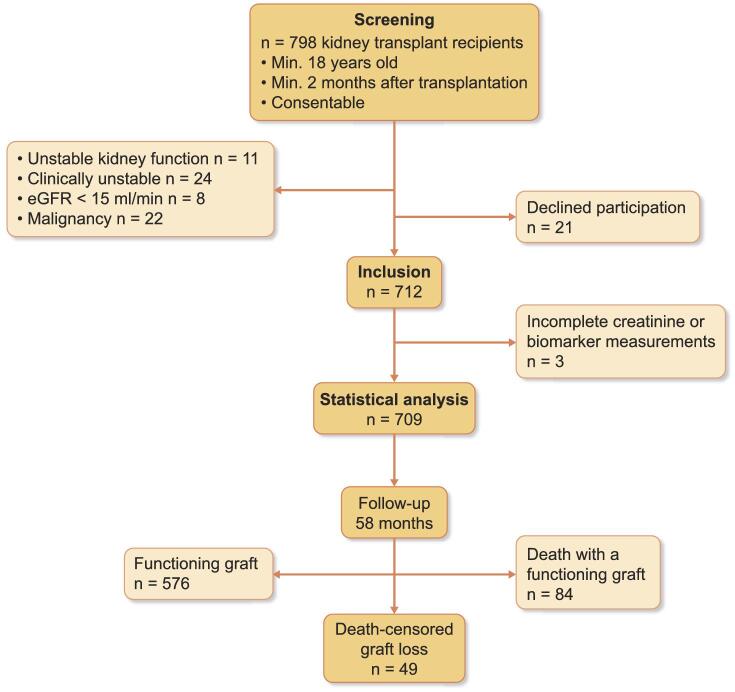
Study flow chart. Enrollment was between 10 April 2013 and 10 September 2013, end of observation was 1 April 2018.

For this monocentric, prospective observational study, all stable patients who were regularly visiting the kidney transplant outpatient clinic of Charité Universitätsmedizin Berlin, Campus Mitte for follow-up care, and who were at least 18 years old, provided informed consent and had received their last kidney allograft more than 2 months ago were eligible for study inclusion. Of 798 potential patients considered for enrollment, 21 refused participation and 65 did not meet inclusion criteria (Fig. 1). A total of 712 patients were enrolled. Three enrolled patients were excluded because of incomplete sample collection at inclusion. Therefore, all analyses were conducted on a total of 709 patients. Figure 1 shows the study flow chart. No patients were lost to follow-up.

The 709 KTRs were predominantly male (60%), on average 54 years old (SD ± 14.35) with a median time since transplantation of 5.4 years (IQR 2.23–10.13). The median follow-up time was 58 months (IQR 57–59). Forty-nine (6.9%) patients had a death-censored graft loss. Table 1 presents demographic data, primary diseases, comorbidities and transplant characteristics. Patients with graft loss were significantly older, had more often received transplants of an extended criteria donor and had more often a history of DGF, antibody-mediated rejection and cell-mediated rejection compared with patients with graft survival. Age of transplant did not differ significantly. Regarding comorbidities, patients with graft loss suffered significantly more often from diabetes, chronic heart failure and coronary artery disease. Patients with graft loss more often received steroids, azathioprine and belatacept as part of immunosuppressive therapy (see Table 1 for details).

**Table 1:** Baseline characteristics at study inclusion.

	**Total cohort**	**Graft loss (death-censored)**	**No graft loss**	***P***
Total number, *n*	709	49 (6.9%)	660 (93.1%)	
Demographics				
** **Age, years, mean (SD)	54.04 (±14.35)	60.63 (±13.12)	53.55 (±14.3)	***.001***
** **Caucasian, *n* (%)	701 (98.9)	48 (98)	653 (98.9)	*.531*
** **Female, *n* (%)	285 (40.2)	17 (34.7)	268 (40.6)	*.415*
Transplantation data				
** **Time since transplantation, years, median (IQR)	5.4 (2.23–10.13)	6.1 (2.53–8.59)	5.4 (2.1–10.28)	*.676*
** **Living donor, *n* (%)	224 (31.6)	11 (22.4)	213 (32.7)	*.139*
** **Expanded criteria donor, *n* (%)	228 (32.2)	30 (61.2)	198 (30)	***<* *.001***
** **DGF, *n* (%)	226 (31.9)	23 (47.9)	203 (32)	***.024***
** **DSA positive at inclusion, *n* (%)	51 (7.2)	10 (21.3)	41 (6.4)	***<* *.001***
** **History of ABMR, *n* (%)	25 (3.5)	10 (21.3)	15 (2.3)	***<* *.001***
** **History of CMR, *n* (%)	209 (29.5)	22 (44.9)	187 (28.3)	***.015***
Immunosuppressive therapy, *n* (%)				
** **Cyclosporine A	274 (38.6)	14 (28.6)	260 (39.4)	*.133*
** **Tacrolimus	329 (46.4)	20 (40.8)	309 (46.8)	*.416*
** **MMF/MPA	659 (92.9)	44 (89.9)	615 (93.2)	*.372*
** **Azathioprine	12 (1.7)	3 (6.1)	9 (1.4)	***.013***
** **Everolimus or rapamycin	65 (9.2)	6 (12.2)	59 (8.9)	*.439*
** **Belatacept	16 (2.3)	4 (8.2)	12 (1.8)	***.004***
** **Steroids	309 (43.6)	33 (67.3)	276 (41.8)	***<* *.001***
History of underlying kidney disease, *n* (%)				
** **Polycystic kidney disease	109 (15.4)	10 (20.4)	99 (15.0)	*.311*
** **Glomerulonephritis	88 (12.4)	8 (16.3)	80 (12.1)	*.389*
** **Hypertensive nephropathy	55 (7.8)	4 (8.2)	51 (7.7)	*.921*
** **Diabetic nephropathy	20 (2.8)	6 (12.2)	14 (2.1)	***<* *.001***
** **Others	437 (61.6)	21 (42.9)	416 (63.0)	***.005***
Comorbidities, *n* (%)				
** **Diabetes mellitus	160 (22.6)	19 (38.8)	141 (21.4)	***.005***
** **Hypertension	691 (97.5)	49 (100)	642 (97.3)	*.242*
** **Coronary heart disease	134 (18.9)	16 (32.7)	118 (17.9)	***.011***
** **Peripheral arterial occlusive disease	63 (8.9)	6 (12.2)	57 (8.6)	*.392*
** **Heart failure	297 (41.9)	34 (69.4)	263 (39.8)	***<* *.001***
** **Cerebrovascular disease	63 (8.9)	5 (10.2)	58 (8.8)	*.737*

*P*-values are indicated for comparison of the differences between patients with graft loss and without graft loss regarding baseline characteristics (Mann–Whitney U test, Student's *t*-test or χ^2^ test as appropriate). Bold values denote statistical significance at the *P* < 0.05 level.

Expanded criteria donors = brain-dead donors ≥60 years old or between 50 and 59 years old with at least two of the following criteria: history of arterial hypertension, last serum creatinine >1.5 mg/dL or cerebrovascular death.

DGF = need for at least one postoperative dialysis in the first 7 days post-transplantation.

Coronary heart disease = history of myocardial infarction, coronary bypass surgery and/or coronary angioplasty.

Heart failure = any degree of insufficiency.

ABMR, antibody-mediated rejection; CMR, cell-mediated rejection; MMF, mycophenolate mofetil; MPA, mycophenolic acid.

### Biomarker levels at enrollment

**Figure 2:**
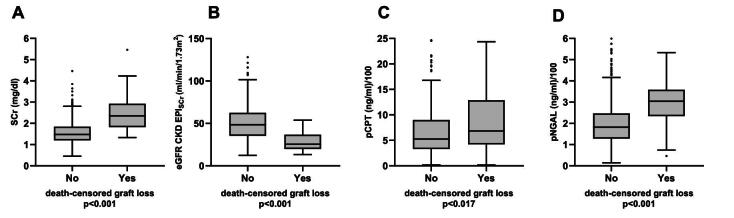
Concentrations of sCr (**A**), eGFR CKD-EPI_sCr_ (**B**), pCPT (**C**) and pNGAL (**D**) at baseline in patients who did or did not experience death-censored graft loss. Boxes show median and lower and upper quartiles. The boundary above and below the boxes indicates data within 1.5 times the IQR. The black dots are outliers. The *P*-value was calculated using Mann–Whitney U test. For better illustration, pNGAL and pCPT values are devided by 100.

Table 2 shows kidney biomarker levels and proteinuria at enrollment. pNGAL, uNGAL, pCPT and sCr levels were significantly higher and eGFR CKD-EPI_sCr_ significantly lower in patients who developed graft loss compared with patients who did not develop graft loss. Urinary CPT (uCPT) levels did not differ significantly between both groups (*P *= .736). Proteinuria (>30 mg/dL by urinary dipstick) was significantly more frequent in patients who later experienced graft loss (63.3% vs 19.8%; *P *< .001). Figure 2 shows the comparison of plasma biomarker levels and eGFR according to graft survival.

**Table 2:** Biomarkers and laboratory parameters at enrollment.

	**Total cohort**	**Graft loss (death-censored)**	**No graft loss**	***P***
Total number, *n* (%)	709	49 (6.9)	660 (93.1)	
pNGAL at inclusion, ng/mL, median (IQR)	189 (130–257)	304 (234.5–358)	182 (128–246)	***<* *.001***
uNGAL at inclusion, ng/mL, median (IQR)	29 (12–57.75)	52 (22–141.5)	28 (12–55)	***.001***
pCPT at inclusion, ng/mL, median (IQR)	539.5 (335.4–913.8)	658.3 (421.5–1287.26)	527. 5 (330.2–902.2)	***.017***
uCPT at inclusion, ng/mL, median (IQR)	57.6 (13.1–301.6)	76.5 (17.3–238.3)	55.7 (13.1–302.8)	*.736*
sCr at inclusion, mg/dL, median (IQR)	1.53 (1.22–1.92)	2.35 (1.82–2.92)	1.48 (1.2–1.84)	***<* *.001***
eGFR at inclusion, mL/min/1.73 m^2^, median (IQR)	46.6 (34.1–61.6)	25.6 (19.9–36.7)	48.5 (35.4–62.4)	***<* *.001***
Dipstick proteinuria at inclusion (≥30 mg/dL), *n* (%)	159 (22.4%)	31 (63.3%)	128 (19.8%)	***<* *.001***

*P*-values are indicated for comparisons of the difference between patients with graft loss and without graft loss (Mann–Whitney U test for continuous variables; χ^2^ test for proteinuria). Bold values denote statistical significance at the *P* < 0.05 level.

IQR = interquartile range.

### Discriminative ability

**Figure 3:**
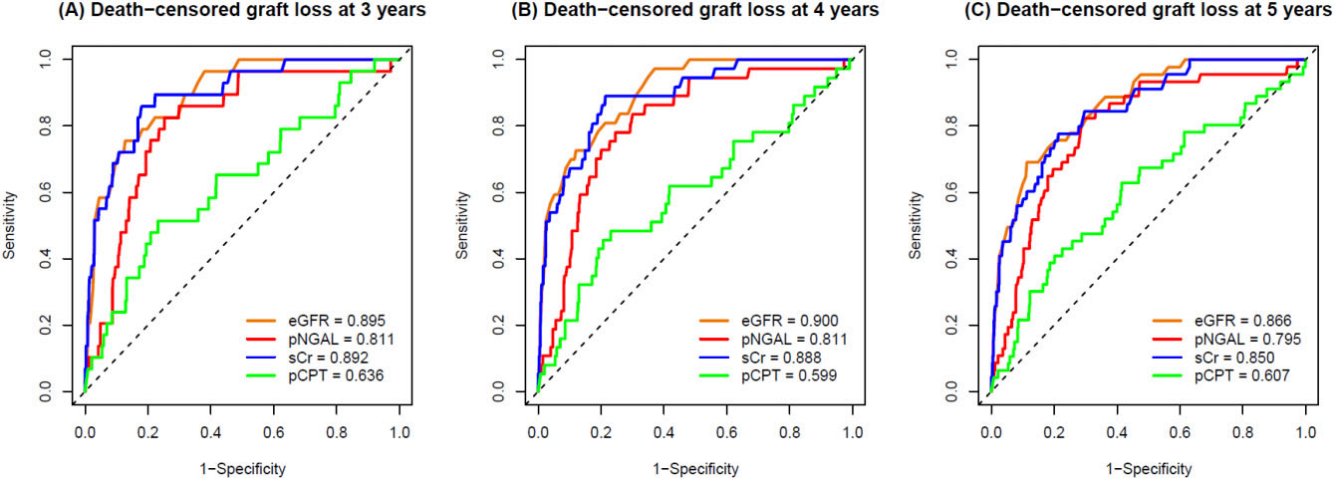
*time*ROC curves and AUC values of eGFR CKD-EPI_sCr_ (orange), pNGAL (red), sCr (blue) and pCPT (green) in predicting death-censored graft loss at 3 (**A**), 4 (**B**) and 5 years (**C**). Reference line (black dashed) indicates AUC = 0.5.

To evaluate the performance of pNGAL and pCPT to predict graft loss after a 3-, 4- and 5-year follow-up in comparison with sCr and eGFR CKD-EPI_sCr_, *time*ROC analyses were conducted. Figure 3 shows ROC curves and AUC for graft loss for each plasma biomarker and eGFR and follow-up period. See Supplementary data, Table S1 for *time*ROC AUC of urinary biomarkers (uNGAL, uCPT).

### Prediction and competing risk models

In the prediction of death-censored graft loss, the best performance by *time*ROC AUC was found for pNGAL, sCr and eGFR CKD-EPI_sCr_. Therefore, we limited the presentation of further analyses to these parameters.

Competing risk analyses were conducted with death as competing risk as described above.

The global correlation tests with Schoenfeld residuals gave no hints for non-proportional hazards for the models presented (see tests results in Supplementary data, Table S2).

Table 3 presents multivariate pNGAL- and eGFR-assisted models adjusted for conventional risk factors for graft loss.

**Table 3:** Multivariate pNGAL and eGFR assisted competing risk model for graft loss with death as competing risk.

	**pNGAL-assisted multivariate model, sHR (95% CI)**	***P***	**eGFR-assisted multivariate model, sHR (95% CI)**	***P***	**pNGAL and eGFR-assisted multivariate model, sHR (95% CI)**	***P***
pNGAL, per doubling	3.23 (1.93–5.41)	<.0001			1.63 (0.92–2.88)	.095
eGFR, per doubling			0.18 (0.10–0.30)	<.0001	0.23 (0.13–0.42)	<.0001
Age, per year	1.04 (1.01–1.06)	.006	1.01 (0.99–1.04)	.308	1.02 (0.99–1.05)	.186
Transplant age, per year	0.99 (0.93–1.04)	.628	1.0 (0.95–1.04)	.915	0.99 (0.94–1.05)	.771
DSA-positive at inclusion	3.08 (1.38–6.91)	.006	3.31 (1.48–7.39)	.004	3.29 (1.51–7.15)	.0034
Proteinuria (≥30 mg/dL)	4.33 (2.34–8.02)	<.0001	3.23 (1.67–6.25)	.0005	3.2 (1.67–6.13)	.0005
History of DGF	1.41 (0.78–2.56)	.257	1.24 (0.65–2.35)	.519	1.3 (0.69–2.45)	.4224

sHR: subdistribution hazard function considers the cumulative incidence in those subjects who are either currently event‐free or who have previously experienced a competing event.

**Figure 4:**
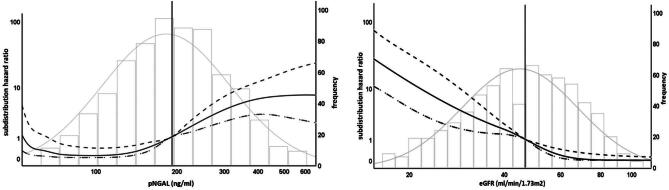
Association of biomarker levels with graft loss and death as competing risk. The left figure shows sHR (left *y*-axis) compared with median pNGAL level (Ref. 189 ng/mL, vertical line), solid line shows unadjusted sHR for pNGAL as spline, dotted lines show upper and lower 95% CIs, histogram shows frequency of pNGAL-levels (right *y*-axis). The right figure shows sHR (left *y*-axis) compared with median eGFR level (Ref. 46. 6 mL/min/1.73 m^2^, vertical line), solid line shows unadjusted sHR for eGFR as spline, dotted lines show upper and lower 95% CIs, histogram shows frequency of eGFR values (right *y*-axis). Curve ends are truncated (value range pNGAL 60–580 ng/mL, eGFR 17–104 mL/min/1.73 m^2^) to avoid overfitting due to small case numbers and wide CIs at the limits of value ranges, logarithmic scales for left *y*-axis and *x*-axis for better differentiation of curves.

In univariate and multivariate biomarker-assisted analyses, there was a strong independent relationship between log2(pNGAL) and risk of graft loss [univariate sub-distribution hazard ratio (sHR) log2(pNGAL) 3.4 (95% CI 2.24–5.15), *P *< .0001; multivariate sHR 3.23, 95% CI 1.93–5.41, *P *< .0001]. The association was substantially attenuated when eGFR was added to the pNGAL assisted-model. Compared with log2(eGFR), proteinuria and DSA, the effect size of log2(pNGAL) was small and did not reach formal significance (*P *= .095) (Table 3). In addition, we performed an exploratory analysis comparing the associations of pNGAL and eGFR with the sHR of graft loss using spline transformation suggesting a potentially more complex association of pNGAL with the outcome (Fig. 4). In fact, spline-transformed pNGAL [spl(pNGAL)] contributed significantly to the multivariate model, independently of eGFR (*P* < .0001).

The bias-adjusted C-statistic for the log2(eGFR)-assisted multivariate model was 0.854 (95% CI 0.803–0.904) implying a good model fit. Adding log2(pNGAL) to the model did not significantly change the C-statistic (0.844, 95% CI 0.791–0.897). Similarly, adding spline-transformed pNGAL instead of log2(pNGAL) to the log2(eGFR)-assisted multivariate model resulted in a C-statistic (0.863, 95% CI 0.815–0.912) without a statistically significant difference to the log2(eGFR)-assisted model (difference: 0.01, 95% CI –0.01 to 0.03).

Since risk modeling and differences in C-statistic may not display sufficient sensitivity in identifying an improvement in individual risk prediction [41, 42], we further preformed an exploratory category-free and two-category (0–5%, >5%) NRI analysis at a 4-year follow-up for a log2(pNGAL)- and log2(eGFR)-assisted multivariate model compared with a log2(eGFR)-adjusted multivariate model. The category-free NRI for events was 31.7% (95% CI −0.52% to 62.9%) and the NRI for non-events was 22.6% (95% CI –3.7% to 48.8%), yielding an overall category-free NRI of 54.3% (95% CI 9.2%–99.3%). The two-category NRI for events was 2.5% (95% CI –5.5% to 10.4%) and that for non-events was –0.2% (95% CI –2.3% to 1.9%). The overall two-category NRI was thus 2.3% (95% CI –6.0% to 10.6%). Similar results were observed with spline-transformed pNGAL (Table 4). These results suggested that the individual changes in risk between the compared models might be present but are minor and probably not clinically significant.

**Table 4:** Summary of the measures of risk reclassification.

	**Bias-adjusted C-statistic (95% CI)**	**Category-free NRI_events_ (95% CI)**	**Category-free NRI_non-events_ (95% CI)**	**Overall category-free NRI (95% CI)**	**2-Catgegory NRI_events_ 0–5%, >5%, (95% CI)**	**2-Catgegory NRI_non-events_ 0–5%, >5%, (95% CI)**	**Overall 2-category NRI 0–5%, >5%, (95% CI)**
Without pNGAL^a^	0.854 (0.803–0.904)						
With log2 (pNGAL)	0.844 (0.791–0.897)	31.7% (0.52% to 62.9%)	22.6% (3.7% to 48.8%)	54.3% (9.2% to 99.3%)	2.5% (−5.5% to 10.4%)	−0.2% (−2.3% to 1.9%)	2.3% (−6.0% to 10.6%)
With pNGAL as splines	0.863 (0.815–0.912)	52.2% (13.3% to 91%)	19.4% (13.9% to 52.6%)	71.5% (28.2% to 114.9%)	3.8% (−7.4% to 15%)	−0.7% (−3.8% to 2.5%)	3.1% (−8.7% to 14.9%)

^a^With base model already including proteinuria ≥30 mg/dL, presence of DSA, age, transplant age, history of DGF and log2(eGFR).

### Association of pNGAL with clinical and laboratory parameters

To gain a better understanding of determinants of plasma NGAL, we explored the association of demographic data, transplant characteristics, immunosuppressive therapy, comorbidities, kidney function and proteinuria with log2(pNGAL) as a dependent variable using linear regression analyses (Table 5). Patient age, transplant age, expanded criteria donor, sCr, the presence of proteinuria and history of rejection correlated positively with pNGAL concentrations, while living donation and eGFR CKD-EPI_sCr_ correlated negatively with pNGAL levels.

**Table 5:** Determinants of pNGAL.

	**Univariate linear regression**				
**Patient characteristics**	**St. β (95%CI)**	***P***	**Corr. R^2^**	**Spearman's correlation coefficient**	***P***
Age (years)	0.09 (0.02 to 0.16)	0.017	0.007	0.116	.002
Caucasian	0.06 (−0.01 to 0.14)	0.099	0.002	0.057	.129
Female sex	−0.06 (−0.13 to 0.02)	0.131	0.002	−0.058	.121
Time since transplantation (years)^a^	0.24 (0.17 to 0.31)	<0.001	0.011	0.176	<.001
Living donor	−0.11 (−0.18 to −0.03)	0.005	0.010	−0.113	.003
Expanded criteria donor	0.21 (0.13 to 0.28)	<0.001	0.042	0.25	<.001
Delayed graft function	0.07 (−0.005 to 0.15)	0.066	0.011	0.099	.009
DSA positive at inclusion	0.05 (−0.03 to 0.12)	0.215	0.001	0.059	.120
History of ABMR	0.13 (0.05 to 0.2)	<0.001	0.014	0.143	<.001
History of CMR	0.14 (0.06 to 0.21)	<0.001	0.018	0.154	<.001
Cyclosporine A	0.04 (−0.04 to 0.11)	0.318	0.000	0.028	.451
Tacrolimus	−0.07 (−0.15 to 0.001)	0.052	0.004	−0.068	.071
MMF/MPA	0.06 (−0.02 to 0.13)	0.14	0.003	0.057	.129
Everolimus or rapamycin	−0.05 (−0.13 to 0.02)	0.148	0.002	−0.049	.196
Steroids	−0.03 (−0.1 to 0.05)	0.446	−0.001	0.006	.869
Diabetes mellitus	−0.004 (−0.08 to 0.07)	0.912	−0.001	0.017	.644
Cardiovascular disease ^b^	0.05 (−0.02 to 0.12)	0.193	0.001	0.045	.227
sCr at inclusion, mg/dL^a^	0.56 (0.5 to 0.62)	<0.001	0.295	0.626	<.001
eGFR CKD-EPI_sCr_ at inclusion, mL/min/1.73 m^2^	−0.52 (−0.58 to −0.46)	<0.001	0.269	−0.615	<.001
Dipstick proteinuria at inclusion (≥30 mg/dL)	0.21 (0.14 to 0.28)	<0.001	0.052	0.232	<.001

Exploratory linear regression analysis to estimate the association of the indicated variables with pNGAL levels. The dependent variable is binary log-transformed pNGAL.

^a^Variable was transformed with binary logarithm.

^b^includes hypertension, coronary heart disease, peripheral arterial occlusive disease, heart failure, cerebrovascular disease.

ABMR, antibody-mediated rejection; CMR, cell-mediated rejection.

In linear regression analyses, the goodness-of-fit measure R^2^ was found to be weak for all variables, except for eGFR and sCr, which displayed a moderate level of fit.

## DISCUSSION

In this monocentric study of stable KTR, pNGAL levels were predictive of death-censored graft loss with death as a competing risk. In a multivariate model including *a priori*–defined established risk factors (age, transplant age, presence of proteinuria and DSA, history of DGF) pNGAL was independently associated with graft loss. The strength of this association was substantially attenuated by adjustment for eGFR.

Inconsistent results were found in the various attempts to assess the additional benefit of pNGAL in predicting graft loss. While pNGAL added in logarithmic form was not an independent predictor in multivariate model, spline transformation of pNGAL showed a significant and independent association with the outcome, suggesting a non-linear or complex relationship between pNGAL and graft loss. An improvement in the concordance index could not be observed. While we observed a positive and significant overall category-free NRI for a pNGAL-assisted model with the same positive trend in NRI of events and in non-events, a categorical NRI analysis did not confirm these findings. Hence, based on our study we were unable to demonstrate a clinically significant improvement of graft loss risk assessment when pNGAL is measured in stable KTR. Nevertheless, future studies with a higher sample size are warranted to definitively resolve this issue.

The results are deemed exploratory and it would be premature to argue for or against the introduction of pNGAL into clinical practice.

Future studies with a higher sample size are warranted to definitively resolve this issue.

The observed correlation of pNGAL and eGFR may explain the limited predictive performance of pNGAL when combined with eGFR. Linear regression analysis indicated that eGFR was a strong determinant of pNGAL. This observation is consistent with the known renal clearance of NGAL [10].

Previous studies tested the utility of NGAL and CPT in the setting of kidney transplantation. Both NGAL and CPT have utility in predicting DGF and poor short-term graft outcomes at the time of kidney transplantation [43–45]. However, much less is known about the utility of these biomarkers in detecting subclinical kidney transplant injury in apparently stable transplant recipients during their follow-up care. In a study design similar to that of our study, Bansal *et al*. reported an association of elevated urinary NGAL with higher risk of graft loss, cardiovascular outcomes and mortality in 1027 stable KTR with a transplant age of at least 6 months [46]. In contrast to the findings from Bansal *et al*., in our study we observed a poor performance of uNGAL in predicting graft loss. One possible reason for this discrepancy may be that asymptomatic sterile leukocyturia was not defined as an exclusion criterion in our study. Leukocyturia contributes to uNGAL concentrations and might be confounding [47].

In a 1-year follow-up, Kielar *et al*. observed an association of high uNGAL levels in stable KTR more than 12 months post-transplantation with a decrease in eGFR of at least 10% compared with KTR with stable or improving eGFR. uNGAL was an independent predictor of eGFR loss, but the discriminatory ability assessed by AUC ROC was poor. KTR with urinary tract infection at baseline were excluded from the study [48]. In contrast to both studies, we observed superior performance of pNGAL, which, unfortunately, was not analyzed in the studies by Bansal *et al*. and Kielar *et al*.

In a recently published study by Kremer *et al*., consistent with our data pNGAL was associated with an increased risk of graft failure in stable KTRs. The latter association was particularly present in KTR with pre-existent poor graft function [49]. However, additional validation studies would be necessary to confirm these findings.

The pathophysiological relevance of elevated NGAL in transplant patients is of interest. NGAL may indicate subclinical renal tubular injury that may be induced in the setting of ongoing immunological subclinical rejection or in calcineurin inhibitor–associated toxicity, which are specific to the renal transplant population. However, NGAL elevations may also be associated with traditional injury processes observed in the kidney, such as the progression of underlying kidney diseases [50], toxin exposure [51], ischemic injury [52] and others [53–56] Thus, NGAL could be a good marker for a variety of different ongoing injury mechanisms, which ultimately may cause graft loss [6]. Schaub *et al*. demonstrated a connection between elevated uNGAL levels and biopsy-proven tubulitis, interstitial fibrosis and tubulus atrophy 3–6 months after transplantation [57], whereas Kaufeld *et al*. found increased uNGAL levels in patients with biopsy-diagnosed acute tubular injury 6 weeks after transplantation, although this association was lost after 6 months [58].

pCPT and uCPT did not have convincing test characteristics for the prediction of graft loss in our study. To our knowledge, no previous studies have addressed the utility of pCPT in stable KTR so far. In short-term observations after transplantation, Tepel *et al*. showed a relationship between elevated uCPT levels and decreases of GFR [59]. In contrast, Seibert *et al*. did not observe a statistically significant association between uCPT levels and a deterioration of kidney function in stable CKD [50]. Elevated pCPT values may reflect systemic inflammation, as has been described in the context of rheumatoid arthritis or cardiovascular disease [60, 61]. In sum, our data do not support a role of uCPT or pCPT in the prediction of long-term outcomes in the setting of the follow-up care of KTR.

In addition to NGAL and CPT, several other potential biomarkers are plausible candidates for early detection and risk stratification of AKI. One example is the marker kidney injury molecule-1 (KIM-1), an indicator of tubular damage that has shown promise in predicting AKI severity and prognosis [62]. The inflammatory cytokine interleukin-18 [63] has emerged as a valuable biomarker reflecting the inflammatory response associated with AKI. The urinary cell cycle arrest markers tissue inhibitor of metalloproteinases 2 (TIMP2) and insulin-like growth factor-binding protein 7 (IGFBP7) [64] have been widely studied in various clinical settings and have potential implications for AKI management in critical ill patients [65, 66]. Their integration into official guidelines is still evolving and their potential for the prediction of long-term graft loss in stable KTR is not clear.

### Study limitations

Our study has several important limitations. First, this study was monocentric and validation in multicentric studies will be necessary to assess reproducibility in other centers. Second, NGAL was measured at only one time point, and all endpoints of the 3- to 5-year follow-up were analyzed relative to this time point of study inclusion. Third, our patient group was heterogeneous with regard to donor and graft characteristics, representing the real-world situation of our transplant center. Several factors that might correlate with an adverse prognosis for graft survival or affect pNGAL levels, such as *de novo* DSA [6, 67] and particularly biopsy-proven pathological changes in the graft (e.g. signs of calcineurin inhibitor toxicity) [6], were not explored. The study design with patient enrollment at variable time points after transplantation is subject to survival bias and a limitation not easily overcome by our statistical approach. Potentially, a clearly defined time point for biomarker determination posttransplant would allow a more reliable evaluation of the predictive performance of the biomarkers. An additional important limitation of our study was the fact that proteinuria measurements were limited to semiquantitative dipstick assessments, which are potentially error-prone [68]. Nevertheless, urine dipstick represents routine clinical practice during kidney transplant follow-up in many centers. Quantitation of urinary albumin/creatinine ratios would be of additional value [69].

Furthermore, we decided against normalization of urinary biomarker levels to urinary creatinine levels. Collection of timed urine specimens to estimate actual creatinine excretion rates was impractical as patients presented as outpatients. As normalization is controversial, this point may be considered a limitation of our study [70, 71].

Finally, despite a large number of patients included, the number of graft losses was limited. Consequently, statistical models with a large number of independent variables (particularly including the set of dummy variables produced for the spline transformation) might tend to instable results.

## CONCLUSION

In this prospective cohort study including 709 stable KTR, we demonstrated that a single pNGAL measurement in the routine follow-up of transplant recipients was predictive for graft loss but did not outperform the predictive ability of the conventional marker eGFR and did not show consistent added value on top of a baseline eGFR-assisted model. Future studies are warranted before implementing recommendations for or against testing pNGAL in clinical practice.

## Supplementary Material

gfad226_Supplemental_File

## Data Availability

The data that support the findings of this study are available from the corresponding author, K.M.S.-O., upon reasonable request.
