# Supplementary material for: Plasma NGAL levels in stable kidney transplant recipients and the risk of allograft loss
Source: Nephrol Dial Transplant. 2023 Oct 19;39(3):483–95. doi: 10.1093/ndt/gfad226 (PMC11024820; doi:10.1093/ndt/gfad226)
Supplement: gfad226_Supplemental_File [file gfad226_Supplemental_File.docx]

# Supporting information

| **Follow-up** | **AUC for uNGAL** | **AUC for uCPT** |
| --- | --- | --- |
| 3 years | 0.705 | 0.544 |
| 4 years | 0.691 | 0.554 |
| 5 years | 0.636 | 0.510 |

Supplemental Table 1: ***Time-dependent Receiver Operating Characteristics (timeROC) Area under Curve (AUC) for graft loss at 3,4- and 5-years*** *with death as competing risk for uNGAL and uCPT. Controls defined as subjects that are free of any event.*

|  | ***Χ^2^*** | **p** |
| --- | --- | --- |
| **log2(pNGAL) assisted multivariate model** | 6.456 | 0.374 |
| **log2(eGFR) assisted multivariate model** | 8.821 | 0.184 |
| **log2(pNGAL) and log2(eGFR) assisted multivariate model** | 8.879 | 0.261 |
| **spl(pNGAL) and log2(eGFR) assisted multivariate model** | 9.657 | 0.562 |

Supplemental Table 2: ***Results of correlation tests for Schoenfeld residuals addressing the proportional hazards assumption****. For this purpose, we used simplified analyses where incomplete data vectors have been deleted and the competing event “death for other reasons” has been considered as censoring. For each presented model, the Chisquare statistic for the global correlation test is presented with corresponding p-value. All model variants met the proportional hazards assumption.*
